# Historical abiotic events or human-aided dispersal: inferring the evolutionary history of a newly discovered galaxiid fish

**DOI:** 10.1002/ece3.1409

**Published:** 2015-03-04

**Authors:** Gamuchirai Chakona, Ernst R Swartz, Albert Chakona

**Affiliations:** 1 Department of Ichthyology and Fisheries Science, Rhodes UniversityP. O. Box 94, Grahamstown, 6140, South Africa; 2 South African Institute for Aquatic BiodiversityPrivate Bag 1015, Grahamstown, 6140, South Africa

**Keywords:** Cape Floristic Region, *Galaxias*, interbasin dispersal, mitochondrial DNA, nuclear DNA, phylogeography

## Abstract

Range expansion of obligate freshwater fishes in the Cape Floristic Region (CFR) of South Africa has mostly been attributed to river capture events and confluence of rivers following sea-level regression. The role of low drainage divides and interbasin water transfers has received less attention. This study analyzed mitochondrial and nuclear DNA sequences to assess the processes that could have influenced the phylogeographic patterns of a newly discovered lineage of *Galaxias zebratus* (hereafter *Galaxias zebratus* “Joubertina”) that occurs across two currently isolated river systems close to the Joubertina area in the eastern CFR. Results from both analyses revealed that observed genetic differentiation cannot be explained by isolation between the two river systems. No genetic differentiation was found between the Krom River system and a population from one of the Gamtoos tributaries. Shallow genetic differentiation was found between the Krom and the other Gamtoos populations. Historical river capture events and sea-level changes do not explain the present distribution of *Galaxias zebratus* “Joubertina” across the Krom and Gamtoos River systems. Interbasin dispersal during pluvial periods, recent river capture, or recent human-mediated translocation seems to be the most plausible explanations.

## Introduction

Freshwater taxa tend to display higher levels of genetic structuring among populations than marine taxa, because they generally have smaller effective population sizes and populations are often isolated by terrestrial or marine barriers (Gyllensten 1985; Ward et al. 1994; DeWoody and Avise 2000). Strong genetic subdivisions are especially expected between separate river systems (Bermingham and Avise 1986; Meffe and Vrijenhoek 1988; Unmack 2001). However, some freshwater taxa have a high salinity tolerance and are therefore capable of marine dispersal. Such diadromous species are characterized by extensive geographic ranges and large population sizes and show low levels of genetic differentiation among populations (Waters et al. 2000).

Species that are not tolerant of high salinities can naturally only disperse between isolated river systems through rare events such as river captures, confluence of rivers during lower sea levels, freshwater plumes in estuarine and marine environments, or flooding of low drainage divides (McGlashan and Hughes 2001; Unmack 2001; Wong et al. 2004; Craw et al. 2007; Burridge et al. 2008; Sharma and Hughes 2011). More extreme possibilities include fish being moved across geographic divides by waterspouts or being dropped by aquatic birds. Such types of dispersal have been reported in Australia (Whitley 1972) and West Africa (Reid 1996), but remain poorly understood (Unmack 2001). Unmack (2001) suggested that there are other possibilities for random dispersal of freshwater fishes through accidental movement of eggs across geographic divides on birds’ feet and/or feathers, while certain insects may be capable of moving eggs over short distances. These means of dispersal have, however, never been scientifically documented.

Several studies have inferred the role of river captures (e.g., Burridge et al. 2007; Swartz et al. 2007; Schönhuth et al. 2011), sea-level changes (Bermingham and Avise 1986; Swartz et al. 2007, 2009), and inland drainage connections through flooding during wet periods (Craw et al. 2007; Burridge et al. 2008; Chakona et al. 2013a) to explain the occurrence of closely related lineages in currently isolated river systems. Accumulating evidence also shows the role of anthropogenic influences in promoting isolation (e.g., Meldgaard et al. 2002) or connectivity (e.g., Hughes et al. 2003; Therriault et al. 2005) of freshwater restricted taxa.

Phylogeographic studies can be employed to understand the history of populations between and within drainages and can identify potential biogeographic processes that influence population genetic structure (Avise 2000; Beheregaray 2008; Chakona et al. 2013a,b). Species that occur on either side of potential barriers are useful for assessing processes that shaped population genetic structure. The Cape Floristic Region (CFR) of South Africa presents an ideal area to study processes that influence the evolutionary history of primary freshwater taxa due to its long period of isolation from surrounding areas and complex drainage patterns. Freshwater taxa in the CFR have been influenced by a potentially broad spectrum of events which include uplift of the Cape Fold Mountains (Hendey 1983; Maud 1990; Hattingh 2008), sea-level changes (Tankard 1976; Siesser and Dingle 1981; Dingle et al. 1983), and climatic oscillations (Partridge et al. 1999).

The impact of this complex history on the CFR's freshwater fauna has been studied with the aid of molecular methods (Waters and Cambray 1997; Bloomer and Impson 2000; Swartz et al. 2007, 2009; Chakona et al. 2013a,b). Several unique genetic lineages of *G. zebratus* have been discovered across the CFR (Waters and Cambray 1997; Wishart et al. 2006; Chakona et al. 2013a,b). Only one of these lineages has a wide distribution range across the CFR (Chakona et al. 2013b), while most of them have restricted geographic ranges. This study builds on previous studies by analyzing the genetic structure of one of these newly discovered lineages in the *G. zebratus* Castelnau, 1861 species complex. This lineage, which occurs only in two currently isolated river systems, the Krom and Gamtoos in the eastern CFR (Cambray et al. 1995; Waters and Cambray 1997), will be referred to as *Galaxias zebratus* “Joubertina” in this study, because the headwaters of the river systems drain upland areas adjacent to the town of Joubertina.

**Figure 1:**
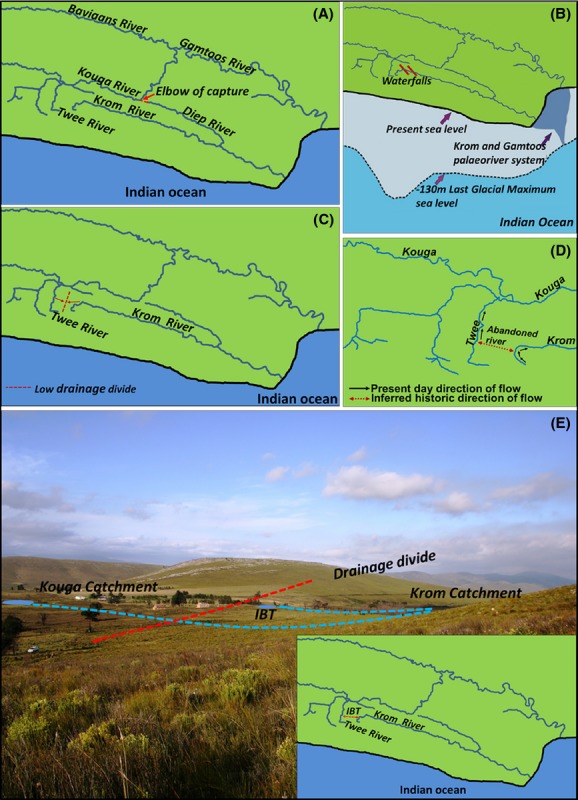
Five mechanisms could have influenced the evolutionary history of the *Galaxias zebratus* “Joubertina” in the Krom and Gamtoos River systems: (A) an ancient Krom-Kouga river capture occurred (Ancient River Capture Hypothesis), (B) confluence of the Krom and Gamtoos river systems during the last glacial maximum (Palaeoriver Hypothesis), (C) low drainage divide that could have been inundated during periods of heavy rainfall (Intermittent Connections Hypothesis), (D) Recent River Capture Hypothesis, and (E) man-made canals between the Gamtoos and Krom River systems (Interbasin Transfer Hypothesis).

*Galaxias zebratus* “Joubertina” seems to be confined to headwater streams of the Krom and Gamtoos River systems, and there has never been a report of this lineage near marine environments or even in main-stem habitats. Marine dispersal capabilities are therefore unlikely. During the Cenozoic (*c*. 60 million years ago), a tributary of the Krom River system (the present day Kouga catchment) was pirated by the Baviaanskloof River, a tributary of the Gamtoos River system (Cambray et al. 1995; Hattingh 2008; Fig.1A). If *Galaxias zebratus* “Joubertina” became isolated *c*. 60 million years ago, one would not only expect deep genetic divergence, but also clear morphological differences observable in the field between populations of the two river systems (Ancient River Capture Hypothesis). Waters and Cambray (1997) found low genetic divergence between the two systems. This finding, coupled with lack of morphological differences, indicates that the Ancient River Capture Hypothesis cannot explain the present distribution pattern of *Galaxias zebratus* “Joubertina”.

Changes in sea levels during the Tertiary (Tankard 1976; Siesser and Dingle 1981; Hendey 1983; Rogers 1985) resulted in periods when connections between river systems in the same valley would have been possible (Swartz et al. 2007, 2009). The Krom and Gamtoos river systems are proposed to have formed a common confluence during the low sea levels of the last glacial maximum (LGM) about 18 000 years ago when the southern African coastline was about 130 m below present day levels. If *Galaxias zebratus* “Joubertina” exploited this connection to disperse between the two river systems, then relatively low levels of genetic differentiation would be expected between populations from the two river systems, with divergence time being consistent with the LGM (Palaeoriver Hypothesis; Fig.1B).

The CFR experienced periods of extreme wet and dry conditions, with the most recent wetter period being inferred to have occurred as recent as the Holocene Altithemal (*c*. 8000 years ago) (Partridge et al. 1999). Temporary periods of integration of the Krom and Gamtoos River systems during periods of heavy flooding could have occurred across the low drainage divide that separates the two river systems. This would be supported by finding shallow or lack of population structuring between the drainages (Intermittent Connections Hypothesis; Fig.1C).

Waters and Cambray (1997) suggested a recent river capture event as a possible explanation for the occurrence of similar cytochrome *b* haplotypes in the Krom and Gamtoos River systems, but the small sample size (one sequence per river system) did not allow them to test this hypothesis. It is possible that the low drainage divide between the Twee and Krom may represent a recently abandoned river course due to river capture (Recent River Capture Hypothesis; Fig.1D).

There are also several man-made dams in the upper catchments of the two river systems (upper Krom and upper Kouga). Recent reports suggest that some of these dams are connected by a series of canals that divert water from the upper Krom to the Kouga catchment (Tweddle et al. 2009). When the dams spill, they discharge into the upper Kouga catchment, establishing a connection between the Krom and Gamtoos River systems. It is possible that *Galaxias zebratus* “Joubertina” could have exploited these man-made canals to disperse between the two river systems (Interbasin Transfer Hypothesis; Fig.1E). This hypothesis would be supported by finding low or no genetic structuring between populations of the two river systems.

The aim of this study was to test these hypotheses to possibly determine how *Galaxias zebratus* “Joubertina” attained its present distribution, specifically its occurrence across two currently isolated river systems.

## Materials and Methods

### Sampling, DNA extraction, and sequencing

**Figure 2:**
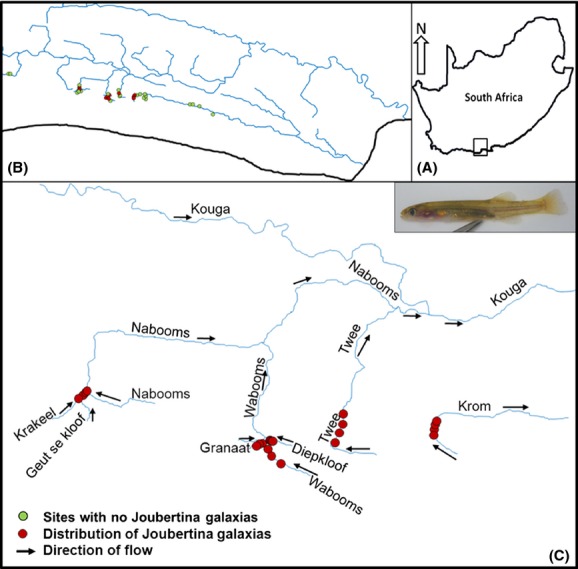
Maps showing the study area (A), sampling sites that were visited to map the distribution of *Galaxias zebratus* “Joubertina” (B) and the present distribution of *Galaxias zebratus* “Joubertina” in the Krom and Gamtoos River systems (C). Arrows in map C indicate the direction of flow.

Samples of *Galaxias zebratus* “Joubertina” were collected from the upper Krom (Krom River system) and upper Kouga (Gamtoos River system) catchments which are located in the Langkloof valley in the Eastern Cape Province of South Africa (Fig.2). Specimens were collected using a 3-m seine net, snorkeling with a hand net, electric fishing, or a combination of these methods. Muscle tissues or whole fish samples were stored in 99% ethanol in the field and, upon returning to the laboratory, were transferred to a −70°C freezer.

Total genomic DNA was isolated from muscle tissue using the Wizard® Genomic DNA purification kit (Promega, USA) following the manufacturer's protocol. A section of the mitochondrial cytochrome *b* gene (*cyt b*) was amplified using the polymerase chain reaction (PCR) with the primers Gcyt-Glu (5′ GAA AAA CCA CCG TTG TTA TTC A – 3′) and Gcyt-Thr (5′ – CGA CTT CCG GAT TAC AAG ACC – 3′) (Waters and Wallis 2001). Amplification was performed using the following thermal profile: initial denaturation at 94°C for 3 min and then 35 cycles of denaturing at 94°C for 30 s, primer annealing at 51°C for 45 s and product extension at 72°C for 50 s, followed by final extension at 72°C for 7 min. Representative subsets of specimens from each mitochondrial clade were amplified for nuclear S7 analysis. The nuclear S7 first intron was amplified using the primers S7RPEX1F (5′ TGG CCT CTT CCT TGG CCG TC – 3′) and S7RPEX3R (5′ – GCC TTC AGG TCA GAG TTC AT – 3′) (Chow and Hazama 1998). S7 amplification was carried out with an initial denaturation at 95°C for 1 min, followed by 30 cycles of amplification (denaturation at 95°C for 30 s, annealing at 60°C for 1 min, and extension at 72°C for 2 min) followed with a final extension at 72°C for 10 min. For both genes, amplification was performed in 25 *μ*L volumes containing 2.5 *μ*L of 10× polymerase reaction buffer, 2.5 *μ*L of 25 mmol/L MgCl_2_, 2.5 *μ*L of 8 mmol/L dNTP's, 0.5 *μ*L of each primer (20 pmol), 0.1 *μ*L of 5 U Super-Therm DNA polymerase (Taq), and 3–5 *μ*L of DNA template (and negative controls with no DNA template to test for contamination). Volumes were adjusted to a final volume of 25 *μ*L with ddH_2_O. All PCR products were purified and sequenced by Macrogen Inc. (South Korea).

### Sequence editing, alignment, and genetic analysis

A total of 121 *cyt b* sequences and 36 S7 sequences were generated for this study. Sequences were checked manually and edited using SeqMan (DNASTAR Lasergene 9 Core Suite). Corrected sequences were aligned using ClustalX (Thompson et al. 1997). Unique and shared haplotypes as well as haplotype frequency (N) per population were identified using DnaSP ver 5.10.01 (Rozas et al. 2003). Within-population variation, using haplotype diversity (*H*_D_) (Nei 1987) and nucleotide diversity (*π*) (Nei and Tajima 1981; Nei 1987), and their standard errors were calculated using DnaSP. A substitution model of sequence evolution that best fitted the data was selected for both genes using Modeltest ver 3.7 (Posada and Crandall 1998). For both genes, genetic differentiation between populations was evaluated by calculating model-corrected pairwise genetic distances between haplotypes in PAUP (Swofford 2002). Divergence times between populations of the Krom and the Gamtoos were estimated using a variable molecular clock, which was recently calibrated for New Zealand galaxiids by Craw et al. (2008).

Network analysis was performed to explore evolutionary relationships among haplotypes using TCS 1.21 (Clement et al. 2000). TCS uses a statistical parsimony method, which links haplotypes with the smallest number of differences as defined by a 95% confidence criterion (Templeton et al. 1992). Analysis of molecular variance (AMOVA, Excoffier et al. 1992) was performed to assess population genetic structuring using the program Arlequin ver 2.0 (Schneider et al. 2000). Four predefined hierarchical structures were tested for both genes to assess which one explained most of the variation in *Galaxias zebratus* “Joubertina”: (1) two groups were defined to test the river systems, and groups were therefore defined as the populations in the Krom and Gamtoos river systems; (2) four groups were defined according to the catchments, namely the Krakeel, Wabooms (including Granaat and Diepkloof), Twee, and Krom catchments; (3) six groups were defined as the individual tributaries, namely the Krakeel, Wabooms, Granaat, Diepkloof, Twee, and Krom, and finally, (4) two groups were defined differently to the first structure by combining the Twee and Krom populations, because they were sharing haplotypes, and compared to all the other populations in the Gamtoos. The significance of the variance components was determined with 1000 permutations (Excoffier et al. 1992).

Pairwise population Ф_ST_ statistics for both genes were calculated using program Arlequin based on the most appropriate substitution model found in Modeltest. Tajima's D (Tajima 1989) and Fu's FS (Fu and Li 1993; Fu 1997) tests for neutrality were performed and tested for significance using 1000 permutations. These tests were performed to determine whether experienced recent range expansion according to the Intermittent Connections hypothesis, Recent River Capture Hypothesis, and/or the Interbasin Transfer hypothesis. Significantly negative values for these tests may also suggest population expansion (Tajima 1989).

## Results

### Distribution

Specimens of *Galaxias zebratus* “Joubertina” were collected from five different tributaries of the Gamtoos River system, namely the Granaat (*N* = 30), Krakeel (*N* = 25), Diepkloof (*N* = 30), and Wabooms (*N* = 30) (referred to in this study as the western Kouga tributaries) and the Twee (*N* = 30) in the eastern part of the Kouga catchment. The species was only found in one tributary of the Krom River system, namely the upper Krom (*N* = 40). Two of these tributaries represent new distribution localities (Krakeel and Diepkloof) (Fig.2C), and the lineage therefore occurs in six tributaries and four broader catchments namely the Krom, Krakeel, Twee, and Wabooms (grouping the three upper Wabooms tributaries, namely the Granaat, Diepkloof, and Wabooms). Specimens were collected from three localities (*N* = 10 per locality) each in the Granaat and Wabooms and four localities in the Krom (10 specimens per locality). Low numbers of individuals were found in the remaining tributaries. To reduce the impact on these populations and to increase the probability of capturing more of the population's genetic diversity, four localities each were sampled for the Twee (2, 8, 10, and 10 specimens per locality, respectively), Krakeel (2, 5, 8, and 10 specimens per locality, respectively), and Diepkloof (5, 5, 6, and 14 specimens per locality, respectively) tributaries. Overall, *Galaxias zebratus* “Joubertina” was only found in 22 localities of the 72 localities that were surveyed (31%).

### Mitochondrial cytochrome *b*

#### Diversity

Analysis of 121 individuals for 616 base pairs of cytochrome *b* resulted in seven haplotypes defined by nine variable sites (Table1). No insertions or deletions were detected, and no significant deviations from those expected under predictions of neutrality were observed when all haplotypes were analyzed together (Tajima 1989; *D *=* *0.016, *P* > 0.10; Fu and Li 1993; *F *=* *0.816, *P *>* *0.10).

**Table 1:** The geographic distribution of mitochondrial cytochrome *b* alleles. Sample sizes (*n*) used in this study and haplotype diversity (*H*_D_) are given at the bottom of the table.

Haplotype	Krom	Twee	Diepkloof	Wabooms	Granaat	Krakeel
Mitochondrial cytochrome *b*						
1	20	20				
2			20	11	8	1
3				8		
4					7	
5				1		
6					6	14
7						5
*n*	20	20	20	20	21	20
*H*_D_	0.000	0.000	0.000	0.563	0.695	0.468
Nuclear S7 diversity						
1	13	14	3	6	8	2
2	1					
3			3	6	2	10
4		2				
5			2			
*n*	14	16	8	12	10	12
*H*_D_	0.143	0.233	0.750	0.545	0. 356	0.468

All the individuals from the Twee and Krom populations shared haplotype 1, but did not share this haplotype with any individuals from other populations. The Diepkloof also had only one haplotype (haplotype 2), but shared it with all the western Kouga tributaries (Diepkloof, Wabooms, Granaat, and Krakeel). Haplotype 6 was restricted to the Granaat and Krakeel tributaries (Table1). There were private haplotypes in the Wabooms (haplotype 3 and 5), Granaat (haplotype 4), and Krakeel (haplotype 7), which were not shared between any of the tributaries. Haplotype diversity (*H*_D_) ranged from 0.468 to 0.695 for the Wabooms, Granaat, and Krakeel, but was 0 for the Krom, Twee, and Diepkloof populations.

The best substitution model selected for cytochrome *b* using MODELTEST was the HKY model (Hasegawa et al. 1985). The model allows for different rates of transitions and transversions as well as accommodating unequal base composition (unequal frequencies of the four nucleotides). Genetic distances between populations of *Galaxias* sp. Joubertina from the Krom and Gamtoos river systems based on the HKY model ranged from 0 to 0.65% (mean 0.37%). Divergence between the Krom and Twee compared to the western Kouga populations ranged from 0.2 to 0.7%. The largest divergence was between the Wabooms and Granaat populations (0–1%) and the Wabooms and Krakeel populations (0–1%). Estimates of divergence times between the Krom and Gamtoos populations ranged between zero and 33 000 years ago. There were no significant differences in gene diversity between the Wabooms, Granaat, and Krakeel populations, but the first two showed relatively higher values compared to the Krakeel population (Table2). Nucleotide diversity was low overall (*π *= 0.003) within populations and did not differ significantly between the Wabooms, Granaat, and Krakeel populations (Table2).

**Table 2:** Mitochondrial cytochrome *b* pairwise Ф_ST_, nucleotide diversity (*π*), and gene diversity (*δ*) values (below diagonal) and nuclear S7 pairwise Ф_ST_, nucleotide diversity (*π*), and gene diversity (*δ*) values (above diagonal) for *Galaxias zebratus* “Joubertina” populations. All pairwise Ф_ST_ comparisons were statistically significant (*P* < 0.05), except for the comparison between the Krom and the Twee. Nucleotide diversity (*π*) and gene diversity (*δ*) values did not differ significantly among the populations.

Population	Krom	Twee	Diepkloof	Wabooms	Granaat	Krakeel	*π*	*δ*
Krom		0.042^NS^	0.416	0.082	0.398	0.754	0.000	0.143
Twee	0^NS^		0.401	0.094	0.377	0.720	0.001	0.233
Diepkloof	1.000	1.000		0.200	−0.006	0.050	0.002	0.750
Wabooms	0.641	0.641	0.354		0.099	0.530	0.001	0.356
Granaat	0.645	0.645	0.260	0.318		0.152	0.001	0.546
Krakeel	0.906	0.906	0.862	0.675	0.511		0.001	0.303
*π*	0.000	0.000	0.000	0.003	0.002	0.001		
*δ*	0.000	0.000	0.000	0.563	0.695	0.468		

NS, not significant.

#### Population genetic structuring within and between populations

**Figure 3:**
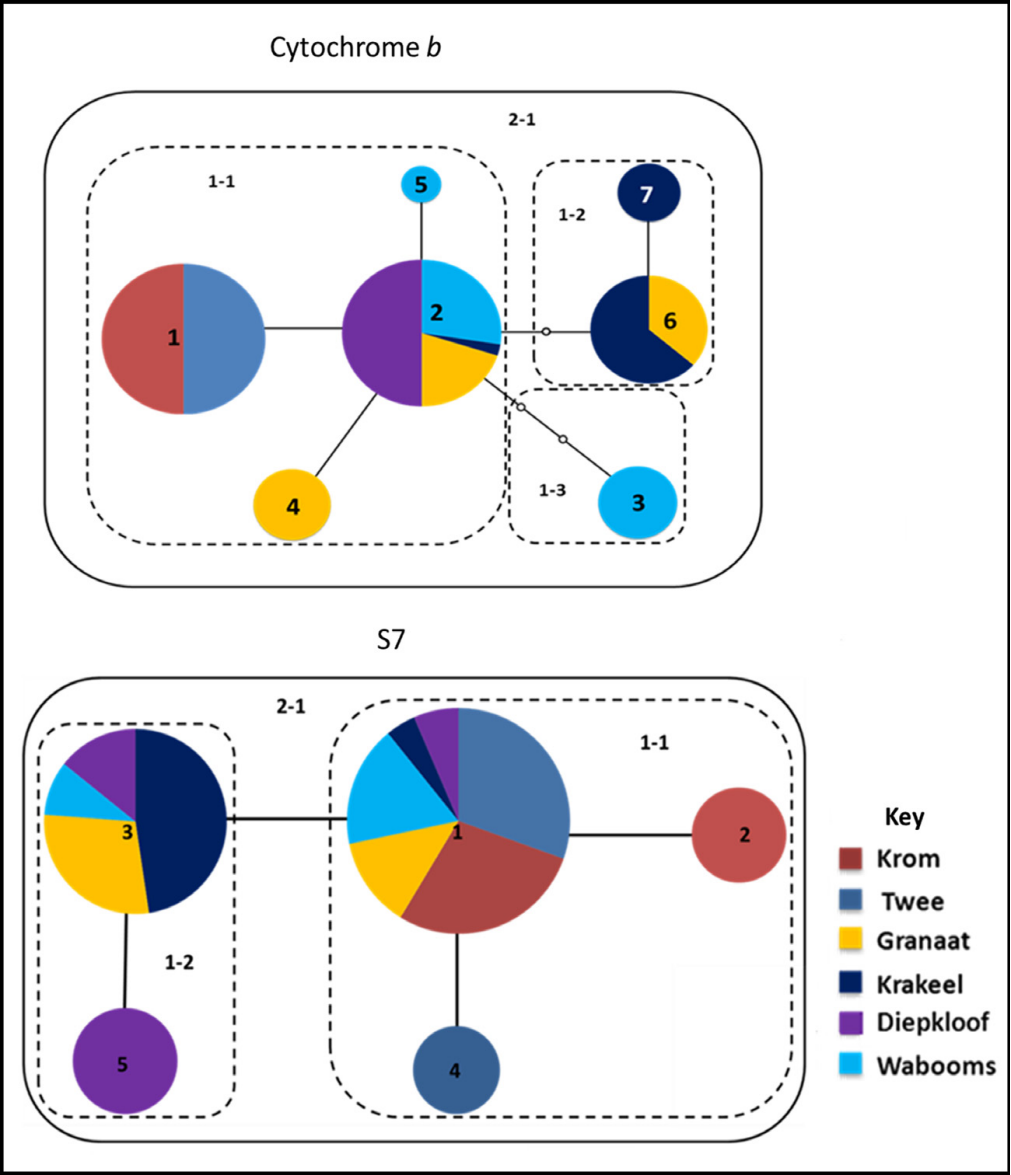
TCS haplotype networks for mtDNA cytochrome *b* (top) and nuclear S7 (bottom) for *Galaxias zebratus* “Joubertina” collected from the Krom and Gamtoos River systems in the Cape Floristic Region, South Africa. Colors correspond to localities where lineages were collected.

Haplotype 2 was the most widespread of the seven haplotypes, occurring in all the tributaries of the Gamtoos River system, except the Twee River (Fig.3). However, three of the Gamtoos tributaries, namely the Granaat, Krakeel, and Wabooms, have unique haplotypes. Results of pairwise population Ф_ST_ statistics revealed significant levels of differentiation for all pairwise population comparisons, except between the Krom and Twee (Table2), which suggest restricted gene flow among all the western Kouga populations. Apart from the lack of sharing of haplotypes between the Krom and Twee compared to all other populations, pairwise Ф_ST_ values revealed significant differentiation among the Krakeel, Granaat, Wabooms, and Diepkloof populations (0.250 < Ф_ST_ < 0.862) (Table2).

AMOVA provided further evidence of structuring among populations of *Galaxias* sp. Joubertina with much of the genetic variation being partitioned among tributaries (*F*_CT_: 0.653 (65.3%) and *F*_SC_: 0.689 (68.9%), *P* < 0.001) (Table3). However, there was no significant differentiation between the river systems, as this level of partitioning explained less than 1% of the variation (*F*_CT_: −0.005 (−0.5%)). This result was due to the sharing of the same haplotype between individuals from the Twee and Krom populations. The overall Ф_ST_ values were all significantly large for all four structures suggesting high levels of differentiation (Table3).

**Table 3:** AMOVA analysis of the distribution of mitochondrial cytochrome *b* genetic variation for populations of *Galaxias zebratus* “Joubertina” based on drainage structure (tributaries, catchments, river systems, and Krom and Twee vs western Kouga tributaries), showing F-statistics (*P *<* *0.05) and percentage variation in brackets.

Source of variation	Six tributaries	Four catchments	Two systems	Twee-Krom vs. W. Kouga
Among groups	0.653 (65.3%)	0.491 (49.1%)	−0.005 (−0.5%)	0.393 (39.3%)
Among populations within groups	0.347 (12.0%)	0.444 (22.6%)	0.686 (68.9%)	0.577 (35.1%)
Within populations	0.773 (22.7%)	0.717 (28.3%)	0.684 (31.6%)	0.743 (25.7%)
Overall *φ*_ST_	0.947	1.060	0.951	1.170

### Nuclear S7

#### Diversity

Analysis of 36 individuals for 492 base pairs of S7 revealed 5 unique haplotypes defined by 4 variable sites. Only one heterozygotic position was found; therefore, 72 sequences were used for analysis after separating the maternally and paternally derived sequences. No significant deviations from those expected under predictions of neutrality were observed when all S7 haplotypes were analyzed together (Tajima (1989): *D *=* *−0.630, *P* = 0.312; Fu and Li (1993): *F *=* *−1.301, *P = *0.223).

Haplotype 1 was found in all six tributaries, but haplotype 3 was restricted to the western Kouga tributaries and was absent in the Twee population. Haplotypes 2, 4, and 5 were restricted to the Krom, Twee, and Diepkloof rivers, respectively (Table1). Haplotype diversity (H_D_) ranged from 0.143 (Krom) to 0.750 (Diepkloof), and overall haplotype diversity was 0.512. Gene diversity was high for the Granaat (0.546 ± 0.062) and Diepkloof (0.750 ± 0.097) populations compared to the other four populations (Table2). Nucleotide diversity was not significantly different among any of the populations, ranging between 0 and 0.002. Overall nucleotide diversity was also low (*π* = 0.001) (Table2).

#### Population genetic structuring within and between populations

Modeltest identified F81 (Felsenstein 1981) as the model that best fitted the S7 data. Model-corrected genetic distances between the Krom and Gamtoos river systems were low and ranged between 0% and 0.4%. Distances between the Twee and western Kouga tributaries ranged between 0 and 0.3%. Results of the variable molecular clock revealed that the divergence between the Krom and Gamtoos haplotypes occurred relatively recently (about 0–19 800 years ago), coinciding with the very recent, Holocene or Pleistocene periods.

**Table 4:** AMOVA analyses for the distribution of nuclear S7 genetic variation for populations of *Galaxias zebratus* “Joubertina” based on drainage structure (tributaries, catchments, river systems and Krom and Twee vs western Kouga tributaries), showing F-statistics and percentage variation in brackets.

Source of variation	Six tributaries	Four catchments	Two systems	Twee-Krom vs. W. Kouga
Among groups	0.305 (30.5%)	0.280 (28.0%)	0.000 (0.01%)	0.362 (36.2%)
Among populations within groups	0.378 (26.3%)	0.194 (14.0%)	0.396 (39.6%)	0.486 (12.4%)
Within populations	0.568 (43.2%)	0.419 (58.1%)	0.396 (60.4%)	0.194 (51.4%)
Overall *φ*_ST_	0.310	0.322	0.310	0.364

All values were statistically significant.

Results of pairwise Ф_ST_ and exact test values (Table2) revealed significant levels of differentiation between the Krom and the Granaat, Wabooms, and Diepkloof tributaries. The highest Ф_ST_ value was observed between the Krom and Krakeel populations, while low Ф_ST_ values were observed between the Krom and Granaat, Wabooms, and Diepkloof populations. The Krom and Twee populations are not genetically differentiated. Pairwise Ф_ST_ values were significant for all comparisons, except only between Krom and Twee populations (Table2). AMOVA analyses indicated that much of the genetic variation was partitioned among tributaries, with no significant differentiation between the two river systems (Table4), confirming the mtDNA results. Variation among populations within groups ranged from 14 to 39.6%. The overall Ф_ST_ values were significantly low suggesting low levels of structuring.

#### Phylogeographic structuring

Haplotype 1 that is central to the network is also the most common haplotype and was found in all six populations. It forms a single clade (clade 1-1) with haplotypes 2 and 4, which are restricted to the Krom and Twee tributaries, respectively. Clade 1–2 is restricted to the western Kouga tributaries and comprises of haplotype 3 (shared among all the western Kouga populations) and haplotype 5 (restricted to the Diepkloof tributary) (Fig.3).

## Discussion

It is generally expected that primary freshwater fish will display high levels of genetic differentiation among populations from isolated river systems, because dispersal opportunities are limited (Ward et al. 1994; McGlashan and Hughes 2001). The lack of genetic differentiation in *Galaxias zebratus* “Joubertina” between the Gamtoos and Krom river systems suggests either that these river systems have not been isolated for a long period of time or that there have been unusual opportunities for dispersal. Populations from the Krom and Gamtoos river systems showed shallow genetic divergence as might be expected for diadromous species, which typically have large population sizes and high levels of gene flow (e.g., Waters et al. 2000; Wong et al. 2004). There is, however, no evidence at this stage to suggest that *Galaxias zebratus* “Joubertina” is diadromous. It is therefore likely that another mechanism allowed occurrence of this lineage in both systems.

The shallow genetic differentiation and recent divergence time estimate (about 0–33,000 years ago for cytochrome *b* and 0–19,800 years ago for S7) between the Krom and Gamtoos populations of *Galaxias zebratus* “Joubertina” are consistent with the proposed connection of the Krom and Gamtoos River systems during the last glacial maximum (LGM) about 18,000 years ago (Swartz et al. 2007). The present Krom population is, however, isolated from the lower reaches of the Krom River system by two large waterfalls (approximately 4–6 m high) that would have prevented upstream movement. This could still have allowed unidirectional gene flow from the upper Krom to the Gamtoos tributaries if there were no barriers in the latter. In addition, Swartz et al. (2009) suggested that the historical isolation of the Krom and Gamtoos populations of *Pseudobarbus afer* was either due to isolation by a barrier during lower sea levels that is now flooded, or it is possible that the Krom was never part of the St. Francis palaeoriver system, forming a separate palaeoriver system during the LGM. If *Galaxias zebratus* “Joubertina” was present in the Krom at the time, it probably did not disperse through proposed palaeoriver systems, because of its absence from middle and lower reach tributaries of both the Gamtoos and Krom river systems. If dispersal occurred between the river systems through downstream areas, individuals would have passed presently unoccupied tributaries and should have established in them. Even if dispersal never occurred, it is nonetheless surprising that *Galaxias zebratus* “Joubertina” has not recently colonized the lower tributaries of both the Krom and Gamtoos river systems from upstream sources. This suggests that lower altitude tributaries might not have suitable habitat, unless the occurrence of *Galaxias zebratus “Joubertina”* in the Krom is so recent that alien fish have prevented downstream colonization. Given the in-stream barriers to dispersal and the absence of *Galaxias zebratus* “Joubertina” in lower altitude tributaries, dispersal through a confluence of the Gamtoos and Krom river systems during the LGM is unlikely and the palaeoriver hypothesis is therefore rejected.

Swartz et al. (2009) proposed inland drainage dispersal during flooding events to explain the lack of divergence between *Pseudobarbus asper* populations from the Gourits and Gamtoos river systems. This may be the case for the genetic similarities between populations of *Galaxias zebratus* “Joubertina” from the Krom and the Gamtoos river systems. There is a low-gradient area between the upper Krom and upper Kouga catchments, which could have facilitated temporary connection of these rivers and dispersal of this lineage during flooding during pluvial periods such as the Holocene Altithermal (*c*. 8000 years ago). Dry conditions, which prevailed after the Holocene Altithermal, could have caused the dissociation of low-gradient connections between the river systems, leaving too little time for loss of diversity or divergence of haplotypes. It is also possible that the low drainage divide was once the drainage line for the Twee flowing into the upper Krom catchment. Although possible, it seems unlikely that the Krom would have flowed into the Kouga catchment, because the upper Krom flows into a deeply incised gorge below the present distribution of *Galaxias zebratus* “Joubertina”. The genetic similarity between fish from the adjacent Twee and Krom compared to the western Kouga tributaries is consistent with the “Intermittent Connections hypothesis.”

Recent geological evidence suggests that the landscape around the areas encompassed by the present study (Kouga and Bainskloof) has undergone remarkably little change for a very long period of time, probably since the Miocene (Bierman et al. 2014). Based on the evidence that the landscape has been relatively unaltered for such a long period of time, the “Recent River Capture Hypothesis” is unlikely to adequately explain the observed genetic patterns and occurrence of *Galaxias zebratus* “Joubertina” in two isolated river systems. Such drainage re-arrangements would need to have occurred over the timescales relevant to the present phylogeographic patterns (which coincides with the Holocene and Peistocene). Alternatively, the lack of differentiation between the Krom and Twee populations could be an indication of ongoing exchange of individuals between the two populations across the drainage divide between the Gamtoos and Krom river systems due to the constructed canals that connect the upper Krom and upper Kouga catchments. The lower reaches of the Twee and the lower sections of the canals are not suitable for the permanent occurrence of the *Galaxias zebratus* “Joubertina,” because of the occurrence of *Micropterus salmoides*, habitat destruction, and unsustainable water abstraction. However, these canals could allow gene flow between the Krom and Twee populations during winter high flows or floods. The Interbasin Transfer Hypothesis can therefore also not be rejected.

The direction of movement between the river systems is uncertain, but there are two possible scenarios that most likely explain why recent gene flow has occurred between these two systems. Firstly, haplotypes may have evolved from a common ancestral haplotype in the Gamtoos River system. The Twee River population would have become isolated relatively recently from the other Gamtoos populations, and a mutation would have caused a unique mitochondrial cytochrome *b* haplotype to evolve in the Twee River. The nuclear data do not show clear isolation in the Twee (although there is a single private haplotype), but this could be due to insufficient time for mutations to accumulate. Connection of the rivers through the Interbasin Transfer (IBT) canal system, natural low-gradient areas, or recent river capture could have then allowed colonization of the Krom River. Alternatively, the Krom was connected to some or all Gamtoos populations during wet periods and later became isolated following onset of contemporary dry conditions. Assuming the Twee population went extinct or never existed in the first place, a unique haplotype could have become isolated in the Krom, which later established in the Twee through dispersal across the IBT, low-gradient connections, or recent river capture. The first scenario seems to be the most plausible explanation, because *Galaxias zebratus* “Joubertina” does not occur elsewhere in the Krom River system, but it occurs in five tributaries of the Gamtoos River system. It is therefore unlikely that the Twee tributary did not have *Galaxias zebratus* “Joubertina” when the Krom population became established. The most likely scenario is that the occurrence of this lineage in the Krom is due to the series of canals (IBT) that connect the two river systems, but the present data cannot reject the possibility of intermittent dispersal across low gradients during wet periods. Both nuclear and mitochondrial DNA revealed relatively higher levels of genetic structuring between *Galaxias zebratus* “Joubertina” populations within the Gamtoos River system. The geographic distribution of genetic diversity within river systems can be influenced by stream structure, physical barriers, isolation by distance, and historical processes on population structure (Shaw et al. 1994; Lu et al. 1997; McGlashan 2000; McGlashan et al. 2001). Isolation by distance is expected when the dispersal distance of an individual is less than the range of the species (Slatkin 1993; McGlashan and Hughes 2001; Sharma and Hughes 2011). *Galaxias zebratus* “Joubertina” showed high levels of differentiation between the Twee and western Kouga populations and between the Krakeel and the three populations of the Wabooms catchment which could be due to isolation by distance. In-stream barriers have been demonstrated to be effective in affecting population genetic structure (Currens et al. 1990; McGlashan and Hughes 2000). High levels of differentiation between the Granaat and the other Gamtoos populations could have been influenced by a small waterfall in the Granaat River a few meters above the confluence with Wabooms River. This waterfall acts as a natural barrier preventing upstream movement of individuals from other populations to the upper Granaat. Stream fishes that are unhindered by natural barriers have highly connected populations and show low levels of genetic differentiation, which is probably the case for the Diepkloof and Wabooms populations. High levels of genetic structuring between Gamtoos populations could also suggest that *Galaxias zebratus* “Joubertina” has limited dispersal ability, a small home range, and/or a preference for certain habitat types.

Recent impacts such as the spread of alien fishes, over abstraction of water, and construction of weirs within the Gamtoos river system are causing population fragmentation which can cause loss of diversity, genetic drift, and inbreeding in populations previously connected by gene flow. This may appear like historical low gene flow when the fish were actually able to migrate historically. Loss of genetic diversity through fragmentation by weirs has been demonstrated (Meldgaard et al. 2002), and this could also be the case for the presence of private haplotypes in the Krakeel population which is fragmented by weirs. Overall, all populations of the western Kouga are extremely fragmented and isolated by long distances due to unsustainable agricultural activities and presence of alien fishes. The fragmented populations occur in small population sizes.

Genetic structuring within the Gamtoos River system was probably influenced by isolation by distance, physical barriers, a natural tendency to have a small home range facilitated by a preference for upper mountain stream habitats, a natural inability to disperse between catchments, and/or recent alien fishes and human impacts. More detailed assessment of the capabilities of *Galaxias zebratus* “Joubertina” individuals to move between populations, and the use of faster evolving genetic markers such as microsatellites, is required to resolve finer scale genetic patterns and shed more light on the evolutionary history of *Galaxias zebratus* “Joubertina.” Such information will also help provide clearer recommendations to conserve the genetic diversity of this taxon.
